# NN-Poly: Approximating common neural networks with Taylor polynomials to imbue dynamical system constraints

**DOI:** 10.3389/frobt.2022.968305

**Published:** 2022-11-08

**Authors:** Frances Zhu, Dongheng Jing, Frederick Leve, Silvia Ferrari

**Affiliations:** ^1^ Hawaii Institute of Geophysics and Planetology, University of Hawaii, Honolulu, HI, United States; ^2^ Sibley School of Mechanical and Aerospace Engineering, Cornell University, Ithaca, NY, United States; ^3^ Air Force Office of Scientific Research, Arlington, VA, United States

**Keywords:** neural networks, safety, interpretability, polynomial, dynamic systems, prediction

## Abstract

Recent advances in deep learning have bolstered our ability to forecast the evolution of dynamical systems, but common neural networks do not adhere to physical laws, critical information that could lead to sounder state predictions. This contribution addresses this concern by proposing a neural network to polynomial (NN-Poly) approximation, a method that furnishes algorithmic guarantees of adhering to physics while retaining state prediction accuracy. To achieve these goals, this article shows how to represent a trained fully connected perceptron, convolution, and recurrent neural networks of various activation functions as Taylor polynomials of arbitrary order. This solution is not only analytic in nature but also least squares optimal. The NN-Poly system identification or state prediction method is evaluated against a single-layer neural network and a polynomial trained on data generated by dynamic systems. Across our test cases, the proposed method maintains minimal root mean-squared state error, requires few parameters to form, and enables model structure for verification and safety. Future work will incorporate safety constraints into state predictions, with this new model structure and test high-dimensional dynamical system data.

## 1 Introduction

Neural networks have emerged as general-purpose regression models and revolutionized fields such as computer vision and machine translation, where occasional errors are less likely to jeopardize human lives. To extend deep neural networks (NNs) to dynamic system applications of high consequence while retaining predictive capabilities, neural networks should be verifiable and conform to physical laws. The original dynamical system adheres to physical laws but the exact form of the dynamical system’s transition model is not known. A neural network excels in learning a representation of the transition model but this representation can disagree with physical laws, even within the bounds of training as the learned features are agnostic/uninformed of the physical laws but particularly outside bounds of training due to lack of generalization. Furthermore, measurement (input) uncertainty or disturbance gives rise to a state prediction that is inaccurate; garbage in—garbage out. For example, if a camera obscurant leads to an incorrect state estimate (not propagating position) while other sensors continue to propagate, a physically uninformed model will incorporate anomalous sensor reading in generating a physically infeasible state prediction. Restructuring a neural network or transforming the NN to a different abstraction could ensure that the model output adheres to physical laws as the original system that generated the data does.

An ideal, interpretable solution leverages the power of neural networks and incorporates physics through 1) trust: safety guarantees in the output prediction and algorithm behavior, 2) causality: deriving relationships between the input and the output, and 3) information: analyzing the abstraction learned from the neural network and inferring system characteristics from the learned parameters and relationships (Lipton, 2018). Trust, or safety, can come in the form of adhering to constraints set by the user to mitigate the impact of a disturbance spike in the input signal on the model prediction. Added domain information in the way of physical laws could counter undesirable behavior. Furthermore, adding constraints has the added benefit that the user imposing some user-defined information into the prediction; that is, there is some component of that system that the user can explain and guarantee in behavior. Causality may be captured by tracing the contribution of an input to output, for which causality in a set of linear equations is very straightforward to correlate contribution from the coefficient matrix in analysis, whereas nesting layers within a neural network is less obvious in drawing input/output casual relationships (saliency maps). Information can include the number of parameters/terms in a model, the familiarity of bases, and the existence of another abstraction: “Computing a tractable function model from the original model can also be viewed as a form of knowledge distillation from the verification perspective, as the function model should be able to produce comparable results or replicate the outputs of the target neural network on specific inputs” (Huang et al., 2019).

This article’s contribution is to solve the system identification or state prediction problem by constructing a mapping from an NN function to a polynomial function from which users can more easily infer behavior (information and causality) and handle changes in constraints (trust). The proposed method approximates a trained NN function of various common architectures (fully connected perceptron, convolution, and recurrent) into a system of linear differential equations in polynomial basis space. As neural networks are universal approximators, the derivation assumes that the general NN structure approximates the system mapping well (Hornik et al., 1989; Pinkus, 1999). The final form of the representation used to approximate the NN is a set of matrix equations with polynomial entries. Constraints or invariant quantities derived from physical laws may then be applied to offer safety and viability to the system dynamics. The polynomial approximation of neural networks is a straightforward mapping, executes in real-time, requires minimal data storage, and limits overfitting by limiting the polynomial expression power. Both the neural network and polynomial abstraction increase the interpretability of the dynamic system that generated the data.

Linear models, the simplest polynomial, are a favored abstraction over neural networks by scientists for their familiarity and analytic traceability. A polynomial basis is capable of expressing nonlinear relationships with a linear model. Polynomials are more computationally tractable, which enables verification (Sidrane et al., 2022). Their analytic traceability enables theoretical guarantees, inference rules (Dutta et al., 2019), and analysis (Lyapunov stability and coefficient stability). In polynomial space, users may apply safety criteria or domain knowledge in the context of constraints, invariant physical quantities, input/output relations, and continuity or bounded sensitivity properties (Narasimhamurthy et al., 2019). The polynomial form appears in many physical contexts (energy, heat transfer, and friction), which may be applied to the approximation as domain knowledge or safety constraints, and could inform scientists of the underlying physical system characteristics.

There is value in obtaining both the universal (NN) approximation and a polynomial representation. Polynomials and NNs with polynomial activation functions are not universal approximators like NNs with sigmoid or tanh activation functions are (Hornik et al., 1989). A generic dataset generated by a dynamic system does not guarantee a sum of squares polynomial solution, which is why a neural network universal approximation is necessary in capturing the transition model from the dynamic data (Ahmadi and Parrilo, 2011). Deep NNs train a non-convex optimization problem, where the constraints do not usually make the solution more tractable as the training protocol typically uses a stochastic gradient search in the first place. Turning the mapping for the dynamics into a polynomial imposes the dynamics as a constraint to be semi-algebraic (i.e., *p*(*x*) > = 0 or *p*(*x*) = 0, including many other polynomial constraints). Semi-algebraic optimization has new powerful results (e.g., sum-of-squares and moment sequences) that allow the highly non-convex optimization program to be converted to an iterative set of semi-definite programs (SDPs) that converge to the global minima. This article’s optimization solution provides the initial mapping for the dynamics represented as a polynomial constraint. Tools, like semi-definite programs, make such optimization problems tractable.

The proposed method advances upon previous methods (Ferrari et al., 2013; Ferrari and Stengel, 2005) by deriving a polynomial abstraction of a trained neural network that is capable of incorporating knowledge and constraints into state prediction by solving the end-to-end polynomial function and constraints simultaneously. Outputs, like a state prediction, can be constrained to adhere to guarantees if the function is polynomial form, whereas neural networks traditionally lack that ability (Rudy et al., 2017; Lagaris et al., 1998; Psichogios and Ungar, 1992). Recently, a class of neural networks ingrain physics directly into the neural network structure, like PINNs (Raissi et al., 2019; Wang et al., 2022), Lagrangian/Hamiltonian NNs (Cranmer et al., 2020; Greydanus et al., 2019), neural ODEs (Djeumou et al., 2022), and deep Markov models (Liu et al., 2022). A difference in our proposed work lies in when the imposition of physics occurs: physics-guided NNs during training and NN-Poly post-training. Our work also differs from strictly learning a polynomial directly from data as we learn a polynomial from a neural network. Furthermore, the polynomial that results from this approach does not have to adhere to the properties of a Lyapunov function as is the case in learning homogenous polynomial Lyapunov functions, in that *V*(*x*) does not need to be strictly positive and $\dot{V}\left(x\right)$ does not need to be strictly negative, where V is traditionally a function of energy (Ahmadi and Parrilo, 2011). While for some state definitions and systems, this form may be the most convenient form that is ultimately used, we do not impose that form in our formulation. The constraint equations that are used may not use energy and may use momentum or distance.

The following sections detail the derivation to approximate a neural network model by transforming the trained NN parameters into coefficients of polynomial form. The derivation consists of four general steps detailed in Sections 2–5:

• Section 2 derives a general Taylor series expansion for a vector function in vector domain (i.e., tensor form) from a trained NN model; higher than second-order derivatives are tensor derivatives.
• Section 3 simplifies tensor derivatives and states to matrix and vector form. An important contribution of this article is unfolding the tensor derivatives into a matrix form and the tensor states into a vector form, which results in matrix manipulability and computation savings.
• Section 4 rewrites the general Taylor series expansion, containing tensor derivatives into Taylor series expansion with only matrix and vector form derivatives. The Taylor series expansion with only matrix and vector form derivatives is desirable because modern scientific programming languages are optimized for vectorized computations. The Taylor series expansion can be presented as an expression in a polynomial form, mapping inputs to outputs. Coefficients of each polynomial entry are derived, and the subsequent dynamic system that the NN approximates can be interpreted.
• Section 5 extends the single-layer polynomial approximation to a multi-layer network, resulting in a polynomial that represents an arbitrarily deep network.

The remaining article sections give context as to how to apply this methodology and show results for simulated dynamic systems.

• Section 6 relates physical constraints for Newtonian dynamics to semi-algebraic constraints that can be applied to the output of the polynomial approximated function.
• Section 7 demonstrates how to solve for state prediction simultaneously with the proposed constraints.
• Section 8 analyzes the proposed NN-extracted polynomial method under various cases. Results show high accuracy and efficiency when dealing with test cases; we discuss how to extend NN-Poly to various other cases.

## 2 Problem formulation: Taylor expansion of a neural network

Representing the NN model as a Taylor polynomial involves two steps. First, a Taylor expansion of vector function ***f*** must be derived in state vector domain ***x***. The input to the NN model is the state vector at some time step, ***x***_*k*_. The output of the NN model is either the state vector at the next time step, ***x***_*k*+1_, or the derivative of the state vector, ${\dot{x}}_{k}$ . The expansion includes various dimensions of tensors and redundant multinomial cross terms. Next, the general derivatives of a neural network are derived in tensor form. The two efforts together produce the final polynomial coefficients for the Taylor polynomial. To validate the method and offer context for other methods, the last section offers a comparison of model fidelity and computation to other numerical system identification methods.

Given a vector input ***x*** ∈ *R* ^*m*×1^ and output ***y*** ∈ *R* ^*n*×1^, a function ***f*** maps the input state to the output state ***f*** (***x***): *R* ^*m*×1^ → *R* ^*n*×1^. Assume the function ***f*** (⋅) is a smooth, continuous vector function, where all derivatives with respect to ***x*** exist and are smooth.

$$y=f\left(x\right)$$ (1)

Given the training pairs (***x***, ***y***), a neural network predicts ${\hat{y}}_{NN}$ with a mapping ***f***_*NN*_(*W*, ***b***, ***x***), given in Eq. 2 where the notation of a hat $\hat{\left(\cdot \right)}$ signifies a prediction of the variable. The learned parameters are *W* and ***b*** in the neural network.

$${\hat{y}}_{NN}=f_{NN}\left(W,b,x\right)$$ (2)

A polynomial ***p*** (***x***) in the form of a Taylor expansion approximates the neural network for which the order of the polynomial *d* that approximates the NN is defined by the NN structure (Rolnick and Tegmark, 2017). The polynomial output ${\hat{y}}_{p}$ is given in Eq. 3, where the expression consists of polynomial coefficients \{*a*_0_, *A* ^1^, …, *A* ^*d*^\}.

$${\hat{y}}_{p}≔p\left(x\right)=a_{0}+A^{1}⊙x+\frac{1}{2!}A^{2}⊙x^{2⊗}+\cdots +\frac{1}{d!}A^{d}⊙x^{d⊗}$$ (3)

The goal is to find coefficients *a* of a polynomial expression that minimizes the error relative to the neural network model, defined by the cost function, *C*, given in Eq. 4 where the coefficients are the set *a* = {*a*_0_, *A* ^1^, …, *A* ^*d*^}.

$$C^{*}=\underset{a}{\mathrm{argmin}}\|f_{NN}\left(W,b,x\right)-p\left(x,a,d\right)\|$$ (4)

The polynomial form is a terminal form of the Taylor expansion, given in Eq. 5, where the *k*th partial derivative of function *f* is given by $\frac{\partial^{k}f}{\partial x^{k}}$ , analogously the Jacobian term, $J_{f}^{k}$ . The polynomial terminates at order *d*, and the remaining higher order terms are captured in ***R*** (***x***).

$$\begin{array}{c} f\left(x\right)=f\left(0\right)+\frac{\partial f}{\partial x}⊙x+\frac{\partial^{2}f}{\partial x^{2}}⊙x^{2⊗}+\cdots +R\left(x\right)=f\left(0\right)+J_{f}^{1}\left(0\right)x+\frac{1}{2!}J_{f}^{2}\left(0\right)⊙x^{2⊗}+\cdots +R\left(x\right) \end{array}$$ (5)

The outer product ⊗ is used in this manuscript to exponentiate a vector, for which an exponentiated vector equation example is given in Eq. 6 and the index notation in Eq. 7, adopted from Granados (2015).

$$x^{3⊗}≔x⊗x⊗x$$ (6)

$${\left(x⊗x⊗x\right)}_{\mathrm{ijk}}=x_{i}x_{j}x_{k}$$ (7)

The inner product ⊙ is used in this manuscript to multiply the function derivatives with the exponentiated states, for which index notation is given in Eq. 8, adopted from Granados (2015).

$$J_{f}^{2}⊙x^{2⊗}=\underset{i}{\sum }\underset{j}{\sum }J_{\mathrm{ijk}}^{2}x_{ij}^{2⊗}$$ (8)

The polynomial expression is a vectorial series defined with Jacobian terms and exponentiated state vector terms in Eq. 9, where the expansion is expressed in a summation over *d* + 1 Jacobian terms. For simplicity, the expansion is about the zero state, assuming ***x***_0_ = 0, which simplifies the lower dimension terms. Note the equivalency of Eqs 3, 9.

$$\begin{array}{cc} p\left(x\right) & =\overset{d}{\underset{k=0}{\sum }}\frac{J_{f}^{k}\left(x_{0}\right)}{k!}⊙{\left(x-x_{0}\right)}^{k⊗}\\ & ≔\overset{d}{\underset{k=0}{\sum }}\frac{1}{k!}A^{k}⊙{\left(x-x_{0}\right)}^{k⊗} \end{array}$$ (9)

Although Eq. 5 is elegant and may be able to derive a closed-form expression for the original function, the subsequent derivative terms incrementally increase in dimension, seen in Eq. 10.

$$\begin{array}{cc} f\left(x\right) & =\left[\begin{array}{c} f_{1}\left(x_{0}\right)\\ f_{2}\left(x_{0}\right)\\ \vdots \\ f_{n}\left(x_{0}\right) \end{array}\right]+\left[\begin{array}{cccc} \frac{\partial f_{1}}{\partial x_{1}} & \frac{\partial f_{1}}{\partial x_{2}} & \cdots & \frac{\partial f_{1}}{\partial x_{m}}\\ \frac{\partial f_{2}}{\partial x_{1}} & \frac{\partial f_{2}}{\partial x_{2}} & \cdots & \frac{\partial f_{2}}{\partial x_{m}}\\ \vdots & \vdots & \ddots & \vdots \\ \frac{\partial f_{n}}{\partial x_{1}} & \frac{\partial f_{n}}{\partial x_{2}} & \cdots & \frac{\partial f_{n}}{\partial x_{m}} \end{array}\right]⊙\left[\begin{array}{c} x_{1}\\ x_{2}\\ \vdots \\ x_{m} \end{array}\right]\\ & \;+\frac{1}{2!}\left[\begin{array}{c} \left[\begin{array}{cccc} \frac{\partial^{2}f_{1}}{\partial x_{1}^{2}} & \frac{\partial^{2}f_{1}}{\partial x_{2}\partial x_{1}} & \cdots & \frac{\partial^{2}f_{1}}{\partial x_{m}\partial x_{1}}\\ \frac{\partial^{2}f_{2}}{\partial x_{1}^{2}} & \frac{\partial^{2}f_{2}}{\partial x_{2}\partial x_{1}} & \cdots & \frac{\partial^{2}f_{2}}{\partial x_{m}\partial x_{1}}\\ \vdots & \vdots & \ddots & \vdots \\ \frac{\partial^{2}f_{n}}{\partial x_{1}^{2}} & \frac{\partial^{2}f_{n}}{\partial x_{2}\partial x_{1}} & \cdots & \frac{\partial^{2}f_{n}}{\partial x_{m}\partial x_{1}} \end{array}\right],\ldots ,\left[\begin{array}{cccc} \frac{\partial^{2}f_{1}}{\partial x_{1}\partial x_{m}} & \frac{\partial^{2}f_{1}}{\partial x_{2}\partial x_{m}} & \cdots & \frac{\partial^{2}f_{1}}{\partial x_{m}^{2}}\\ \frac{\partial^{2}f_{2}}{\partial x_{1}\partial x_{m}} & \frac{\partial^{2}f_{2}}{\partial x_{2}\partial x_{m}} & \cdots & \frac{\partial^{2}f_{2}}{\partial x_{m}^{2}}\\ \vdots & \vdots & \ddots & \vdots \\ \frac{\partial^{2}f_{n}}{\partial x_{1}\partial x_{m}} & \frac{\partial^{2}f_{n}}{\partial x_{2}\partial x_{m}} & \cdots & \frac{\partial^{2}f_{n}}{\partial x_{m}^{2}} \end{array}\right] \end{array}\right]⊙\left[\begin{array}{cccc} x_{1}^{2} & x_{1}x_{2} & \cdots & x_{1}x_{m}\\ x_{2}x_{1} & x_{2}^{2} & \cdots & x_{2}x_{m}\\ \vdots & \vdots & \ddots & \vdots \\ x_{m}x_{1} & x_{m}x_{2} & \cdots & x_{m}^{2} \end{array}\right]\\ & \;+\cdots +R\left(x\right) \end{array}.$$ (10)

Instead, we would like a set of linear equations that simply solves for the polynomial coefficients with a single matrix operation, given in Eq. 11. To transform the various tensors into a set of linear equations, the derivative tensor terms must be unfolded and compressed into matrices and vectors to achieve the desired form, given in Eq. 11, described in the following sections.

$$f\left(x\right)=\left[\begin{array}{ccccccccccc} f_{1}\left(x_{0}\right) & \left(\frac{\partial f_{1}}{\partial x_{1}}\right) & \cdots & \left(\frac{\partial f_{1}}{\partial x_{m}}\right) & \left(\frac{\partial^{2}f_{1}}{\partial x_{1}^{2}}\right) & \cdots & \left(\frac{\partial^{2}f_{1}}{\partial x_{1}\partial x_{m}}\right) & \cdots & \left(\frac{\partial^{2}f_{1}}{\partial x_{m}^{2}}\right) & \cdots & \left(R_{1}\left(x^{k⊗}\right)\right)\\ & & & & & \vdots \\ f_{n}\left(x_{0}\right) & \left(\frac{\partial f_{n}}{\partial x_{1}}\right) & \cdots & \left(\frac{\partial f_{n}}{\partial x_{m}}\right) & \left(\frac{\partial^{2}f_{n}}{\partial x_{1}^{2}}\right) & \cdots & \left(\frac{\partial^{2}f_{n}}{\partial x_{1}\partial x_{m}}\right) & \cdots & \left(\frac{\partial^{2}f_{n}}{\partial x_{m}^{2}}\right) & \cdots & \left(R_{n}\left(x^{k⊗}\right)\right) \end{array}\right]\left[\begin{array}{c} 1\\ x_{1}\\ \vdots \\ x_{m}\\ \frac{1}{2!}x_{1}^{2}\\ \vdots \\ \frac{2}{2!}x_{1}x_{m}\\ \vdots \\ \frac{1}{2!}x_{m}^{2}\\ \vdots \\ 1 \end{array}\right]$$ (11)

## 3 Unfolding and compressing tensors into matrices and vectors

**FIGURE 1:**
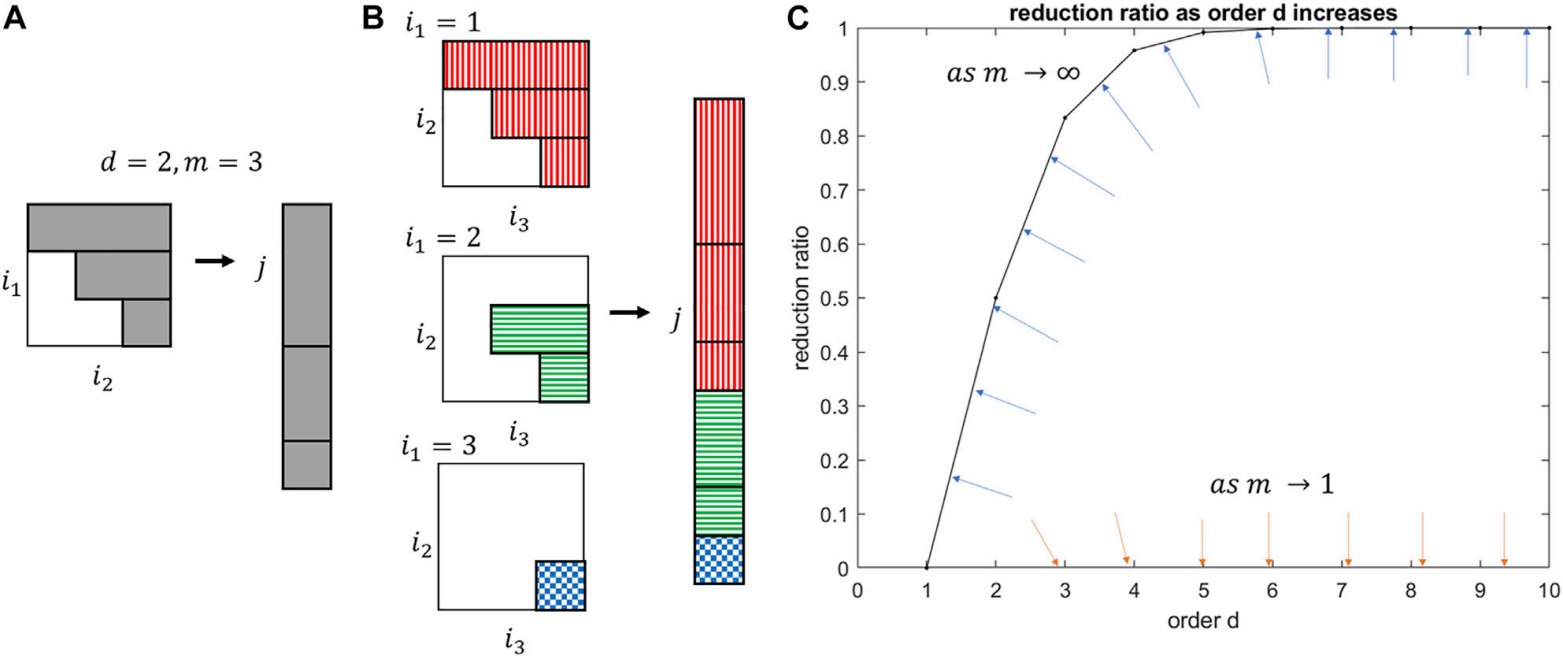
**(A)** Unfolding an upper triangular matrix into a vector. **(B)** Unfolding a tensor into a vector. **(C)** Reduction ratio between number of reductions and number of full terms. The blue and orange arrows represent the movement of the reduction ratio curve at two extremes: blue as the state vector dimension approaches infinity; orange as the state vector dimension approaches one.

The tensor equation that approximates the original function may be collapsed into a set of linear equations, consisting of a coefficient matrix and state vector, a useful form for a linear least square solution or semi-algebraic optimization. Exponentiated states of order higher than two are in the form of tensors and contain redundant multinomial terms as they are symmetric. By analogy, the upper triangular part of a symmetric matrix contains all of its unique values. To unfold and reshape the high dimensional tensors to matrices and vectors, the tensor indices of each higher dimension has a relationship to a single index in the vector and the matrix. The derivation may be intuited visually. The columns of the upper triangular matrix are sequentially appended to a vector, seen in Figure 1A. This process is extended to symmetric tensors, Figure 1B, to yield the augmented state vector.

By grouping redundant multinomial cross terms together into a coefficient vector, the input vector contains only the unique states. The first step is gathering redundant state terms in the same equation, highlighted (Eq. 12). The desired matrix equation, (Eq. 11), of the tensor equation yields the same set of linear equations, yet reduces the number of total state terms and is in a more useful representation. The augmented state vector in Eq. 11 contains scalar coefficients, which represent the number of redundant terms in the multinomial expansion of the state. These scalar coefficients are separated into a new coefficient vector. Defining this new coefficient vector is necessary to reform the exponentiated states into a state vector of only unique states and in the process reveals the reduction in computation by using only unique states. For intuition, the unique multinomial state vector of third order is ${\tilde{x}}^{⊗3}$ , given in Eq. 13, where the addition of the tilde annotates that the uniqueness operation has been performed on the original state vector ***x*** ^⊗3^. The corresponding multinomial coefficient vector, ***a*** ^3^, contains respective coefficients, representing the combinatorial number of redundant multinomial states, given in Eq. 14.

$$f\left(x\right)=\left[\begin{array}{c} \begin{array}{c} f_{1}\left(x_{0}\right)+\left(\frac{\partial f_{1}}{\partial x_{1}}x_{1}+\frac{\partial f_{1}}{\partial x_{2}}x_{2}+\cdots +\frac{\partial f_{1}}{\partial x_{m}}x_{m}\right)+\cdots \\ \;+\frac{1}{2!}\left[\left(\frac{\partial^{2}f_{1}}{\partial x_{1}^{2}}x_{1}^{2}+\frac{\partial^{2}f_{1}}{\partial x_{2}\partial x_{1}}x_{2}x_{1}+\cdots +\frac{\partial^{2}f_{1}}{\partial x_{m}\partial x_{1}}x_{m}x_{1}\right)\right.+\cdots \\ \;\;+\left(\frac{\partial^{2}f_{1}}{\partial x_{1}\partial x_{m}}x_{1}x_{m}+\frac{\partial^{2}f_{1}}{\partial x_{2}\partial x_{m}}x_{2}x_{m}+\cdots +\frac{\partial^{2}f_{1}}{\partial x_{m}^{2}}x_{m}^{2}\right)+\cdots +R_{1}\left(x^{k⊗}\right)\\ f_{2}\left(x_{0}\right)+\left(\frac{\partial f_{2}}{\partial x_{1}}\right.x_{1}+\frac{\partial f_{2}}{\partial x_{2}}x_{2}+\cdots +\left(\frac{\partial f_{1}}{\partial x_{m}}x_{m}\right)+\cdots \\ \;+\frac{1}{2!}\left[\left(\frac{\partial^{2}f_{2}}{\partial x_{1}^{2}}x_{1}^{2}+\frac{\partial^{2}f_{2}}{\partial x_{2}\partial x_{1}}x_{2}x_{1}+\cdots +\frac{\partial^{2}f_{2}}{\partial x_{m}\partial x_{1}}x_{m}x_{1}\right)\right.+\cdots \\ \;\;+\left(\frac{\partial^{2}f_{2}}{\partial x_{1}\partial x_{m}}x_{1}x_{m}\right.+\left(\frac{\partial^{2}f_{2}}{\partial x_{2}\partial x_{m}}x_{2}x_{m}+\cdots +\frac{\partial^{2}f_{2}}{\partial x_{m}^{2}}x_{m}^{2}\right)+\cdots +R_{2}\left(x^{k⊗}\right)\\ \vdots \\ f_{n}\left(x_{0}\right)+\left(\frac{\partial f_{n}}{\partial x_{1}}x_{1}+\frac{\partial f_{n}}{\partial x_{2}}x_{2}+\cdots +\frac{\partial f_{n}}{\partial x_{m}}x_{m}\right)+\cdots \\ \;+\frac{1}{2!}\left[\left(\frac{\partial^{2}f_{n}}{\partial x_{1}^{2}}x_{1}^{2}+\frac{\partial^{2}f_{n}}{\partial x_{2}\partial x_{1}}x_{2}x_{1}+\cdots +\frac{\partial^{2}f_{n}}{\partial x_{m}\partial x_{1}}x_{m}x_{1}\right)\right.+\cdots \\ \;\;+\left(\frac{\partial^{2}f_{n}}{\partial x_{1}\partial x_{m}}x_{1}x_{m}+\frac{\partial^{2}f_{n}}{\partial x_{2}\partial x_{m}}x_{2}x_{m}+\cdots +\frac{\partial^{2}f_{n}}{\partial x_{m}^{2}}x_{m}^{2}\right)+\cdots +R_{n}\left(x^{k⊗}\right) \end{array} \end{array}\right]$$ (12)

$${\tilde{x}}^{⊗3}=\left[\begin{array}{cccccccccccccccc} x_{1}^{3}, & x_{1}^{2}x_{2}, & \cdots \;, & x_{1}^{2}x_{m}, & x_{1}x_{2}^{2}, & \cdots \;, & x_{1}x_{2}x_{m}, & \cdots \;, & x_{1}x_{m}^{2}, & x_{2}^{3}, & x_{2}^{2}x_{3}, & \cdots \;, & x_{2}^{2}x_{m}, & x_{2}x_{m}^{2}, & \cdots \;, & x_{m}^{3} \end{array}\right]$$ (13)

a3=13!3332,1⋯32,131,2⋯31,1,1⋯32,13332,1⋯32,131,2⋯33=13!13⋯33⋯6⋯313⋯33⋯1 (14)

This augmented state vector is the unique multinomial state vector, ${\tilde{x}}^{⊗d}$ , which is $\tilde{x}$ exponentiated to degree *d* in Eq. 15, where *j* is the vector term index and *i*_1_, *i*_2_, …, *i*_*d*_ are the tensor dimension indices.

$$\begin{array}{cc} {\tilde{x}}^{⊗d}\left(j\right) & =x^{⊗d}\left(i_{1},i_{2},\ldots ,i_{d}\right)\\ & =x_{i_{1}}x_{i_{2}}\cdots x_{i_{d}}\\ & \text{where}\;j=i_{1}+\frac{i_{2}\left(i_{2}-1\right)}{2}+\cdots +\overset{d}{\underset{k=1}{\prod }}\frac{i_{d}+k-2}{k}\\ & \;\text{for}\;i_{1}=1:m,\\ & \;\text{for}\;i_{2}=i_{1}:m,\\ & \;\cdots \;,\\ & \;\text{for}\;i_{d-1}=i_{d-2}:m,\\ & \;\text{for}\;i_{d}=i_{d-1}:m \end{array}$$ (15)

$$n_{d}=\overset{d}{\underset{i=1}{\prod }}\frac{m+i-1}{i}$$ (16)

This augmented state vector, ${\tilde{x}}^{⊗d}$ , is of size *n*_*d*_, given in Eq. 16 and calculated with the multinomial theorem (Hildebrand, 2009). Unfolding tensors is a non-unique process, for which a different unfolding yields a different vector of coefficients. These solution sets are all minima for the same unfolding problem. Supplementary Appendix Section S10.1 gives the explicit description of augmented state vectors in ascending degree and corresponding multinomial coefficient vector.

The general solution for the multinomial coefficient vector ***a*** ^*d*^(*j*) is given in Eq. 17, where the operator (⋅) is the binomial coefficient of choosing *d* states out of *m* total number of states and *n*_*i*_ is the number of individual *x*_*i*_ states in the multinomial state ${\tilde{x}}^{⊗d}\left(j\right)$ (Hildebrand, 2009). The size of ***a*** ^*d*^ also follows (Eq. 16). The multinomial coefficient vector index *j* in Eq. 17 aligns with the state *j* index in Eq. 17. Explicit coefficient definitions for ascending orders of ${\tilde{x}}^{⊗d}\left(j\right)$ are given in Table 8 in Supplementary Appendix Section S10.1.

$$\begin{array}{cc} a^{d}\left(j\right) & =\frac{1}{d!}\left(d,n_{1},n_{2},,\ldots ,n_{m}\right)\text{for}\;n_{1}\\ & =O\left(x_{1}\right)\in {\tilde{x}}^{⊗d}\left(j\right),\;\cdots \;,\;n_{m}=O\left(x_{m}\right)\in {\tilde{x}}^{⊗d}\left(j\right) \end{array}$$ (17)

The general solution for the Jacobian matrix ${\tilde{J}}_{f}^{2}\left(:,j\right)$ is given in Eq. 18. The index *j* of the modified Jacobian ${\tilde{J}}_{f}^{d}$ mapping follows the index *j* of the multinomial state vector ${\tilde{x}}^{⊗d}$ mapping with one additional rule: the first dimension’s index *i*_0_ in the original Jacobian tensor $J_{f}^{d}$ directly translates to the first dimension’s index *i*_0_ in the modified Jacobian matrix ${\tilde{J}}_{f}^{d}$ . An example of the explicit definition of ${\tilde{J}}_{f}^{2}\left(:,j\right)$ can be found in Table 8 in Supplementary Appendix Section S10.1. Note that for *d* ≤ *m*, *i*_1_ to *i*_*d*−*m*+1_ does not increment in index but stays at index 1 as dummy dimensions for the multinomial state, coefficient, and Jacobian derivations.

$$\begin{array}{cc} {\tilde{J}}_{f}^{d}\left(i_{0},j\right) & =J_{f}^{d}\left(i_{0},i_{1},i_{2},\ldots ,i_{d}\right)\\ \text{for}\;j & =i_{1}+\frac{i_{2}\left(i_{2}-1\right)}{2}+\cdots +\overset{d}{\underset{k=1}{\prod }}\frac{i_{d}+k-2}{k}\\ \text{for}\;i_{0} & =1:n\\ \text{for}\;i_{1} & =1:m,\\ \text{for}\;i_{2} & =i_{1}:m,\\ \cdots \;,\\ \text{for}\;i_{d-1} & =i_{d-2}:m,\\ \text{for}\;i_{d} & =i_{d-1}:m \end{array}$$ (18)

Compressing redundant state terms saves an immense amount of computation, especially for the number of states in real-world applications and for deriving approximations with more than two derivatives. The general reduction ratio, *r*, between the number of unique terms and the full expansion is given in Eq. 19.

$$r=1-\frac{\prod_{i=1}^{d}\frac{m+i-1}{i}}{m^{d}}$$ (19)

The reduction can be seen to approach 100% for large states and 0% for scalar domain, Figure 1C. The real reduction rate falls somewhere beneath the *m* = *∞* bounding curve and the *m* = 1 origin. The minimum number of states and derivatives to yield a significant computation reduction of 25% is already achieved at *m* = 2 states and *d* = 2 derivatives. Table 6 in Supplementary Appendix Section S10.1 illustrates how the reduction ratio scales with the derivative order, explicitly calculating the corresponding number of states for full state tensor expansion compared to the unique state terms in the augmented state vector ${\tilde{x}}^{⊗d}$ . Motivated by linear solutions and significant computational cost, a framework was derived such that the tensor derivatives and states in the Taylor expansion can be reshaped into a unique multinomial matrix and vectors, respectively.

## 4 Tensor derivatives of a neural network

This section derives different single-layer neural network’s tensor derivatives $J_{f}^{d}$ that populate the Taylor series. The tensor derivatives evaluated at the origin ${\left.J_{f}^{d}\right|}_{x=0}$ are the coefficient tensors *A* ^*d*^. This section’s derivation is the next step in the overall process of approximating a single-layer neural network, with a polynomial function. The derivative formulation covers a wide range of the most popular networks, classified into network type and activation function. Network layers contained in this section are feedforward, convolution, and recurrent layers. Activation functions include binary, max, linear, ReLU, softmax, sigmoid, tanh, and probabilistic, referenced in Table 1.

**TABLE 1:** Activation function type by ascending complexity with their associated vector and index expressions.

Activation function name	Activation function vector expression	Activation function index expression
Binary		$y_{j}=\left\{\begin{array}{cc} 1,\; & \text{if }\sum_{i=1}^{m}w_{ji}x_{i}+b_{j}\geq 0\\ 0,\; & \text{otherwise} \end{array}\right.$
Max	*y* = max(*W* ***x*** + ***b***)	*y*_*j*_ = max(*w*_*ji*_ *x*_*i*_ + *b*_*j*_) for *i* = 1, 2, *…*, *m*
Linear	***y*** = *W* ***x*** + ***b***	$y_{j}=\sum_{i=1}^{m}w_{ji}x_{i}+b_{j}$
Softmax	$y=\frac{e^{Wx+b}}{e^{1\cdot \left(Wx+b\right)}}$	$y_{j}=\frac{e^{\sum_{i=1}^{m}w_{ji}x_{i}+b_{j}}}{\sum_{k=1}^{m}e^{\sum_{i=1}^{m}w_{ki}x_{i}+b_{k}}}$
Probabilistic	$y=e^{-\beta_{i}\|x-c_{i}\|}$	$y_{j}=e^{-\beta_{i}}\sqrt{\sum_{i=1}^{m}{\left(x_{i}-c_{ij}\right)}^{2}}$

**FIGURE 2:**
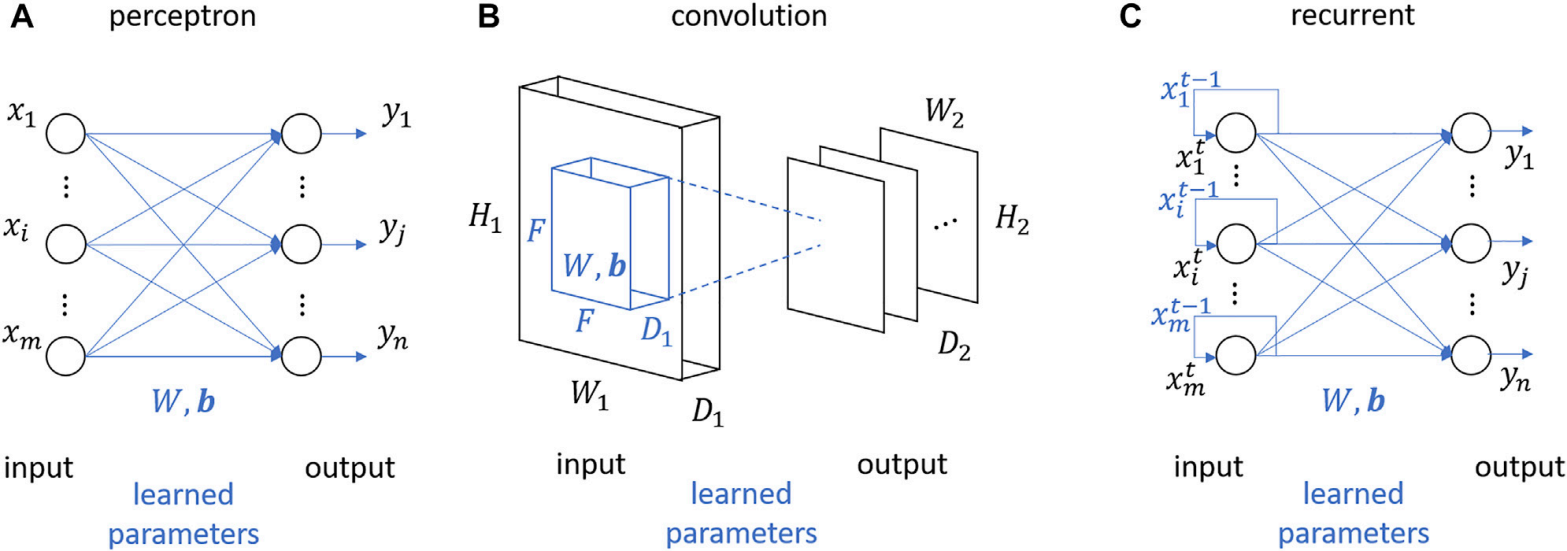
Basis single layer types transforming input to output: **(A)** perceptron layer, **(B)** convolution layer, and **(C)** recurrent layer.

The tensor derivatives that approximate the neural network layer are dictated by the pairing of cell layer type and activation function type; a feedforward layer with a ReLU activation has different tensor derivatives than a feedforward layer with a sigmoid activation. The following subsections vary the relevant transformations *σ*(⋅) and calculates ascending orders of different NN layer derivatives to then populate a Taylor approximation in tensor form (Eq. 5), and then explicitly in matrix form (Eq. 18). The layer types are illustrated in Figure 2.

### 4.1 Perceptron layer

A single-layer feedforward network with *n* number of neurons in the layer is depicted in Figure 2A. The output from a hidden layer ***y***_*F*_ is a transformation ***f***_*F*_ of the input ***x***, and a learned weight and bias matrix, *W* and ***b***. The input and output are in vector form, where ***x*** is of size $R^{m\times 1}$ and ***y***_*F*_ is of size $R^{n\times 1}$ . The vector equation is given in Eq. 20, where *σ*(⋅) is the activation function.

$$y_{F}=f_{F}\left(x\right)=\sigma \left(W,b,x\right)$$ (20)

The index equation is given in Eq. 21, where the order of indices follows the dimension. The index, *i*, corresponds to a value in input state ***x*** and the index, *j*, corresponds to a value in output state ***y***_*F*_.

$$y_{j}=f_{j}\left(x_{i}\right)=\sigma \left(w_{ji},b_{j},x_{i}\right)$$ (21)

For many activation functions, derivatives truncate in finite order, such as binary, linear, and ReLU functions. The coefficient tensors for these terminating derivatives are trivial solutions as their Taylor series are equivalent to their original function expressions. These derivations may be found in Supplementary Appendix Section S10.2. For continuously differentiable activation functions, like the sigmoid, tanh, softmax, and probabilistic, the Taylor approximation series is artificially truncated at a user-defined order *d* (Eq. 22).

$$\begin{array}{cc} & y_{j}\approx a_{j}^{0}+\overset{m}{\underset{i=1}{\sum }}A_{ji}^{1}x_{i}+\frac{1}{2!}\overset{m}{\underset{i_{1}=1}{\sum }}\overset{m}{\underset{i_{2}=1}{\sum }}A_{ji_{1}i_{2}}^{2}x_{i_{1}i_{2}}^{⊗2}+\cdots \\ & \;+\frac{1}{d!}\overset{m}{\underset{i_{1}=1}{\sum }}\cdots \overset{m}{\underset{i_{d}=1}{\sum }}A_{ji_{1}\cdots i_{d}}^{d}x_{i_{1}\cdots i_{d}}^{⊗d} \end{array}$$ (22)

The higher order polynomial coefficient tensors are iteratively derived with immediately previous polynomial coefficient tensors. The general derivative relationships for each activation function are proved by induction from lower order derivatives. The general sigmoid derivative is given in Eq. 23 with the 0th to 2nd order terms given in Eqs 88–90 in Supplementary Appendix as proof of the iterative pattern.

$$A_{ji_{1}\cdots i_{d}}^{d}=\frac{\partial A_{ji_{1}\cdots i_{d-1}}^{d-1}}{\partial \sigma }w_{ji_{d}}\sigma \left(b_{j}\right)\left(1-\sigma \left(b_{j}\right)\right)$$ (23)

The tanh derivative is given by Eq. 24 with 0^*th*^ to 2^*nd*^ derivatives in Eqs 91–93 in the Supplementary Appendix.

$$A_{ji_{1}\cdots i_{d}}^{d}=\frac{\partial A_{ji_{1}\cdots i_{d-1}}^{d-1}}{\partial \sigma }w_{ji_{d}}\left(1-\sigma^{2}\left(b_{j}\right)\right)$$ (24)

The softmax derivative is given by Eq. 25 with 0^*th*^ to 1^*st*^ derivatives in Eqs 94, 95 in Supplementary Appendix.

$$A_{ji_{1}\cdots i_{d}}^{d}=A_{ji_{1}\cdots i_{d-1}}^{d-1}w_{ji_{d}}$$ (25)

Probabilistic activation functions come in many different forms of which the Gaussian or radial basis function is the most popular function, given in Table 1. The polynomial coefficients for this function are complicated expressions for which two intermediate expressions simplify and reveal iterative patterns more clearly in Eq. 26: $\alpha_{t}^{D}$ and $s_{t}^{D}$ . Each polynomial coefficient tensor is a sum *n*_*t*_ terms composed of $\alpha_{t}^{D}$ and $s_{t}^{D}$ , where *t* the increment of a subexpression and *D* is the term order. The number of terms *n*_*t*_ depends only on the term’s degree order D, defined in Eq. 27.

$$A_{ji_{1}\cdots i_{d}}^{d}=\overset{n_{t}}{\underset{t=1}{\sum }}\alpha_{t}^{D}s_{t}^{D}$$ (26)

$$n_{t}=\left\lceil \frac{D+1}{2}\right\rceil$$ (27)

The most general terms, $\alpha_{t}^{D}$ and $s_{t}^{D}$ , for any term and degree definition are in Eqs 28, 29, with explicit definitions in Table 9 in Supplementary Appendix Section S10.2 and 0th to 4th order terms are explicitly given in Eqs 99–103 in Supplementary Appendix.

$$\alpha_{t}^{D}=\overset{t-1}{\underset{k=1}{\prod }}\left(2k-1\right){\left(\frac{-\beta_{j}}{\left\|c_{j}\right\|}\right)}^{t-1}\alpha_{1}^{D-t+1}$$ (28)

$$s_{t}^{D}=\overset{D-2\left(t-1\right)}{\underset{l=1}{\prod }}\left(\overset{D}{\underset{k=l}{\sum }}\frac{\beta_{j}c_{ji_{k}}}{\left\|c_{j}\right\|}\right)$$ (29)

### 4.2 Convolution layer

The convolution layer commonly uses two types of activation functions, linear and max, to achieve filtering and pooling. The derivatives of these activation functions truncate either on the 0th or 1st order derivative. The coefficient tensors when injected into the Taylor series yield exact representations of the original activation functions but offer standard indexing for matrix and vector conversion. A single-layer convolution layer with *n* filters of *f* spatial extent, *s* stride, and *p* zero padding is depicted in Figure 2B. Like the perceptron layer, the output from the hidden layer, *Y*, is a transformation of the input, *X*, and a learned weight and bias tensor, *W* and ***b***. Unlike the perceptron layer, the input and output are typically in the matrix or third order tensor form, where X is of size $R^{w_{1}\times h_{1}\times d_{1}}$ and *Y* is of size $R^{w_{2}\times h_{2}\times d_{2}}$ . The tensor equation is given in Eq. 30.

$$Y=f_{C}\left(X\right)=\sigma \left(W,b,X\right)$$ (30)

The tensor equation with indices is given in Eq. 31. The indices *i*, *j*, *k* for the output range from: *i* = 1, 2, *…*, *w*_2_, *j* = 1, 2, *…*, *h*_2_, and *k* = 1, 2, *…*, *d*_2_. The indices *l*, *m*, *n* range from: *l* = 1, 2, *…*, *w*_1_, *m* = 1, 2, *…*, *h*_1_, and *n* = 1, 2, *…*, *d*_1_.

$$Y_{\mathrm{ijk}}=\sigma \left(W_{ij}^{k},b_{k},X_{\mathrm{lmn}}\right)$$ (31)

The linear activation function within a convolution layer is often called a convolution filter and offers feature extraction of input data. The convolution filter function utilizing the convolution operator in matrix and index form is given in Eq. 32, where the definition of ${\tilde{X}}_{ij}$ is given in Eq. 33.

$$Y_{\mathrm{ijk}}=W^{k}⊛{\tilde{X}}_{ij}+b_{k}$$ (32)

$${\tilde{X}}_{ij}=X\left[s\left(i-1\right)-p+1:s\left(i-1\right)-p+f,s\left(j-1\right)-p+1:s\left(j-1\right)-p+f,1:d_{1}\right]$$ (33)

The relationship between input and output sizes is defined by the network hyperparameters, reiterated here: number of filters *n*, height and width of the filter *f*, stride length *s*, and padding *p*, given in Eq. 34.

$$w_{2}=\frac{w_{1}-f+2p}{s}+1,\;h_{2}=\frac{h_{1}-f+2p}{s}+1,\;d_{2}=n$$ (34)

The hyperparameters *n*, *f*, *s*, *p* are user-defined for which a common setting for the hyperparameters is: *f* = 3, *s* = 1 and *p* = 1. The constraint *p* = (*f* − 1)/2 preserves input to output size. The learned parameters *W* is of size $R^{f\times f\times n}$ and *b* is of size $R^{n}$ . The output equation in strictly index form is given in Eq. 35.

$$Y_{\mathrm{ijk}}=\overset{f}{\underset{l=1}{\sum }}\overset{f}{\underset{m=1}{\sum }}\overset{d_{1}}{\underset{n=1}{\sum }}W_{\mathrm{lmn}}^{k}X_{l+s\left(i-1\right)-p,m+s\left(j-1\right)-p,n}+b_{k}$$ (35)

The linear activation function is a continuously differentiable function with a Taylor approximation series that truncates after the 1st order term, given in Eq. 36.

$$Y_{\mathrm{ijk}}=a_{\mathrm{ijk}}^{0}+\overset{w_{1}}{\underset{l=1}{\sum }}\overset{h_{1}}{\underset{m=1}{\sum }}\overset{d_{1}}{\underset{n=1}{\sum }}A_{\mathrm{ijklmn}}^{1}X_{\mathrm{lmn}}$$ (36)

The 0th and 1st order terms are given in Eqs 37, 38. $A_{\mathrm{ijklmn}}^{1}$ is a sparse six dimensional tensor that performs an equivalent convolution operation with *W* ^*k*^. The Taylor approximation yields an exact representation to the original function expression despite the difference in operation representation.

$$a_{\mathrm{ijk}}^{0}=f_{\mathrm{ijk}}\left(X_{\mathrm{lmn}}=0\right)=b_{k}$$ (37)

$$\begin{array}{cc} A_{\mathrm{ijklmn}}^{1} & ={\left.\frac{\partial f_{\mathrm{ijk}}}{\partial X_{\mathrm{lmn}}}\right|}_{X_{\mathrm{lmn}}=0}\\ & =\left\{\begin{array}{cc} W_{l+s\left(1-i\right)+p,m+s\left(1-j\right)+p,n}^{k},\; & \text{if }1+s\left(i-1\right)+p\leq l\leq f+s\left(i-1\right)+p\\ \; & \text{ and }1+s\left(j-1\right)+p\leq m\leq f+s\left(j-1\right)+p\\ 0,\; & \text{otherwise} \end{array}\right. \end{array}$$ (38)

The maximum activation function within a convolution layer is often called a pooling layer. A pooling layer typically follows a filter layer, offering translation invariance in terms of convolution filter output. The max function outputs the maximum value of the input that falls within the kernel. The matrix form is given in Eq. 39.

$$Y_{\mathrm{ijk}}=\underset{i,j,k}{\max}\left(X\left[s\left(i-1\right)+1:s\left(i-1\right)+f,s\left(j-1\right)+1:s\left(j-1\right)+f,k\right]\right)$$ (39)

As there are no distinct kernels and no padding, the only hyperparameters are field size *f* and stride *s* for which the relationship between input and output sizes is defined in Eq. 40.

$$w_{2}=\frac{w_{1}-f}{s}+1,\;h_{2}=\frac{h_{1}-f}{s}+1,\;d_{2}=d_{1}$$ (40)

Common settings for the hyperparameters are *f* = 2 and *s* = 2. The Taylor approximation follows the same form as Eq. 36, which truncates after the 1st order term. The 0^*th*^ and 1^*st*^ order terms are given in Eqs 41, 42. The Taylor approximation yields an exact representation to the original function expression despite the difference in operation representation.

$$a_{\mathrm{ijk}}^{0}=f_{\mathrm{ijk}}\left(X_{\mathrm{lmn}}=0\right)=0$$ (41)

$$A_{\mathrm{ijklmn}}^{1}=\left\{\begin{array}{cc} 1,\; & \text{if }X_{\mathrm{lmn}}=\max\left({\tilde{X}}_{\mathrm{ijk}}\right)\\ 0,\; & \text{otherwise} \end{array}\right.$$ (42)

### 4.3 Recurrent layer

The structure of a single-layer with *n* number of recurrent units in the layer is depicted in Figure 2C. The output from the convolution hidden layer, $y_{C}^{t}$ or equivalently ***s*** ^*t*^, is a transformation of the current time step’s input, ***x*** ^*t*^, the previous time step’s internal state, ***s*** ^*t*−1^, and a learned weight and bias matrices, *W*, *U*, and ***b***. The input, internal state, and output are typically in vector form, where ***x*** ^*t*^ is of size $R^{m\times 1}$ but ***s*** ^*t*−1^ and $y_{C}^{t}$ are of size $R^{n\times 1}$ . The weights are typically in matrix form, where *W* is of size $R^{n\times n}$ , *U* is of size $R^{n\times m}$ , and ***b*** is of size $R^{n\times 1}$ . The vanilla recurrent unit vector relationship is given in Eq. 43.

$$y=f\left(s^{t-1},x^{t}\right)=\sigma \left(W,s^{t-1},U,x^{t},b\right)$$ (43)

The index relationship is given in Eq. 44, where the order of indices follows the dimension. The index *i* corresponds to a value in the internal state ***s*** ^*t*−1^, the index *k* corresponds to a value in the input state ***x*** ^*t*^, and the index *j* corresponds to a value in the output state $y_{C}^{t}$ .

$$y_{j}=f_{j}\left(s_{i}^{t-1},x_{k}^{t}\right)=\sigma \left(w_{ji},s_{i}^{t-1},u_{jk},x_{k}^{t},b_{j}\right)$$ (44)

The explicit vanilla RNN vector equation and index equation are given in Eqs 45, 46, respectively.

$$y=\sigma \left(Ws^{t-1}+Ux^{t}+b\right)$$ (45)

$$y_{j}=\sigma \left(\underset{i}{\sum }w_{ji}s_{i}^{t-1}+\underset{k}{\sum }u_{jk}x_{k}^{t}+b_{j}\right)$$ (46)

The vanilla RNN vector equation can be reformed into a feed-forward layer with analogous tensor and coefficient derivations. The previous state ***s*** ^*t*−1^ and the current input ***x*** ^*t*^ may be joined together into one input ***z***, given in Eq. 47.

$$z^{t}=\left[s^{t-1}\;x^{t}\right]$$ (47)

The combined input ***z*** is of additive size $R^{\left(n+m\right)\times 1}$ with associated dimension index *l*. The combined weight matrix, *V*, is given in Eq. 48 and is of size $R^{n\times \left(n+m\right)}$ .

$$V=\left[W\;U\right]$$ (48)

The reformed state prediction vector equation and index equation are given in Eqs 49, 50.

$$y_{C}^{t}=\sigma \left(Vz^{t}+b\right)$$ (49)

$$y_{j}=\sigma \left(\underset{l}{\sum }v_{jl}z_{l}+b_{j}\right)$$ (50)

The similarity between the reformed equations of the recurrent layer and feed-forward layer is apparent. To be explicit, the modified Taylor series expansion about the reformed state ***z*** is given in Eqs 51, 52.

$$f\left(z\right)=f\left(z_{0}\right)+J_{f}^{1}\left(z_{0}\right)z+\frac{1}{2!}J_{f}^{2}\left(z_{0}\right)⊗z^{2⊗}+\cdots +R_{n}\left(z\right)$$ (51)

$$y_{j}\approx a_{j}^{0}+\overset{n+m}{\underset{l=1}{\sum }}A_{jl}^{1}z_{l}+\frac{1}{2!}\overset{n+m}{\underset{l_{1}=1}{\sum }}\overset{n+m}{\underset{l_{2}=1}{\sum }}A_{jl_{1}l_{2}}^{2}z_{l_{1}l_{2}}^{⊗2}+\cdots +\frac{1}{d!}\overset{n+m}{\underset{l_{1}=1}{\sum }}\cdots \overset{n+m}{\underset{l_{d}=1}{\sum }}A_{jl_{1}\cdots l_{d}}^{d}z_{l_{1}\cdots l_{d}}^{⊗d}$$ (52)

The tensor and coefficient derivations can be found in Supplementary Appendix Section S10.2] as the form of the equations are identical but the input state, weight matrix, and respective indices are directly analogous to the feedforward derivation.

## 5 Multiple layer network approximation

The output of the multi-layer network is an embedded Taylor approximation of each individual layer. Given a network with *k* layers, the final output ***y*** is a transformation of the *k*th intermediate state ***z*** ^*k*^. The output expression is given in Eq. 53.

$$\begin{array}{cc} y & =f_{o}\left(z^{k}\right)\\ & =a^{k,0}+A^{k,1}z^{k}+A^{k,2}z^{k,⊗2}+\cdots +A^{k,d}z^{k,⊗d} \end{array}$$ (53)

The last hidden layer prior to the output layer transforms intermediate state ***z*** ^*k*−1^ to ***z*** ^*k*^, given in Eq. 54, repeated for all intermediate states *z* for hidden layers 1 to *k* − 1. The input layer transforms the data input ***x*** to the first intermediate state ***z*** ^1^, given in Eq. 55.

$$\begin{array}{cc} z^{k} & =f_{k}\left(z^{k-1}\right)\\ & =a^{k-1,0}+A^{k-1,1}z^{k-1}+A^{k-1,2}z^{k-1,⊗2}+\cdots +A^{k-1,d}z^{k-1,⊗d} \end{array}$$ (54)

$$\begin{array}{cc} z^{1} & =f_{1}\left(x\right)\\ & =a^{i,0}+A^{i,1}x+A^{i,2}x^{⊗2}+\cdots +A^{i,d}x^{⊗d} \end{array}$$ (55)

The mapping from the input to the output is a recursive function embedding intermediate functions backward from the final layer, given in Eq. 56.

$$y=f\left(x\right)=f_{o}\left(f_{k}\left(\cdots f_{2}\left(f_{1}\left(x\right)\right)\cdots \;\right)\right)$$ (56)

A verification of the multi-layer approximation may be found in Supplementary Appendix Section S10.4.

## 6 Physical constraints in the form of semi-algebraic constraints

The development of new semi-algebraic optimization routines motivate a polynomial representation of an unknown dynamical system extracted from NN. Since many physical vector fields are at least piecewise smooth, we can use Taylor’s theorem to construct a polynomial that uniformly approximates the underlying ground truth arbitrarily tightly as the degree increases.

In the context of dynamical systems, physical laws provide valuable context that may be applied to state prediction. A more informed state prediction is found by combining the NN-derived polynomial state equations and user-defined semi-algebraic constraints. This is mainly enticing when learning and updating the new polynomial expression on-the-fly when new data is gathered. Hierarchies of semi-definite and sometimes even, linear program relaxations may be possible toward convergence of a global minimizer within the set of coefficients as parameters for such a problem. Moreover, incorporation of such constraints on the admissible set for its domain may indeed collapse the degree and required state dimension of the learned polynomial. Indeed, after the initial and possibly large polynomial is accurately extracted from the NN differential equation from the analytic unconstrained methods presented in this article, a new optimization goal would be to set up a hierarchy of optimization problems minimizing simultaneously, degrees and state dimensions, thus compressing dimension of the parameter space. The dimension of the parameter space, *n*_*s*_, is given in Eq. 57, where *n* is the size of the state ***s*** and *d* is the degree of the polynomial.

$$n_{s}\left(d\right)=\left(\begin{array}{c} n+d\\ d \end{array}\right)=\frac{\left(n+d\right)!}{d!n!}$$ (57)

This section offers common constraints but in application, any constraint in polynomial form may be defined and appended to this larger set of linear equations. A comprehensive state for rigid body dynamics is translation [*x y z*] and rotation, [*θ*_*x*_ *θ*_*y*_ *θ*_*z*_], in all six degrees of freedom and the associated velocity states, denoted by a derivative dot above the state. The full state vector is shown in Eq. 58.

$$s=\left[x\;y\;z\;\dot{x}\;\dot{y}\;\dot{z}\;\theta_{x}\;\theta_{y}\;\theta_{z}\;{\dot{\theta }}_{x}\;{\dot{\theta }}_{y}\;{\dot{\theta }}_{z}\right]$$ (58)

For a predictive dynamics model, the neural network could be trained to develop a discrete transition model ***f***_***d***_ to yield the next state ***s***_*k*+1_ from the current state *s*_*k*_ or a continuous model ***f***_***c***_ to yield the current state derivative ${\dot{s}}_{k}$ , given in Eqs 59, 60, respectively.

$$s_{k+1}=f_{d}\left(s_{k}\right)$$ (59)

$${\dot{s}}_{k}=f_{c}\left(s_{k}\right)$$ (60)

Some suggested constraints of interest can be separated into systems under no external influence, external influence, and physical constraints. Under no external influence, a system’s total energy (kinetic energy and potential energy *U*) is conserved, given in Eq. 61. Similarly, momentum is conserved in both translation and angular, given in Eq. 62. If rotational degrees of freedom are constrained, the *θ* terms in Eq. 62 are zero, and for constrained translation, the *x*, *y*, *z* terms are zero.

$$\begin{array}{cc} & \frac{1}{2}\left(\left[{\dot{x}}_{k}\;{\dot{y}}_{k}\;{\dot{z}}_{k}\right]M\left[\begin{array}{c} {\dot{x}}_{k}\\ {\dot{y}}_{k}\\ {\dot{z}}_{k} \end{array}\right]+\left[\theta_{xk}\;\theta_{yk}\;\theta_{zk}\right]I\left[\begin{array}{c} \theta_{xK}\\ \theta_{yk}\\ \theta_{zK} \end{array}\right]\right)+U\left(s_{k}\right)\\ & \;=\frac{1}{2}\left(\left[{\dot{x}}_{k+1}\;{\dot{y}}_{k+1}\;{\dot{z}}_{k+1}\right]M\left[\begin{array}{c} {\dot{x}}_{k+1}\\ {\dot{y}}_{k+1}\\ {\dot{z}}_{k+1} \end{array}\right]+\left[\theta_{x,k+1}\;\theta_{y,k+1}\;\theta_{z,k+1}\right]I\left[\begin{array}{c} \theta_{x,k+1}\\ \theta_{y,k+1}\\ \left.\theta_{z,k+1}\right] \end{array}\right]\right)+U\left(s_{k+1}\right) \end{array}$$ (61)

$$M\left[\begin{array}{c} {\dot{x}}_{k}\\ {\dot{y}}_{k}\\ {\dot{z}}_{k} \end{array}\right]+I\left[\begin{array}{c} \theta_{x,k}\\ \theta_{y,k}\\ \theta_{z,k} \end{array}\right]=M\left[\begin{array}{c} {\dot{x}}_{k+1}\\ {\dot{y}}_{k+1}\\ {\dot{z}}_{k+1} \end{array}\right]+I\left[\begin{array}{c} \theta_{x,k+1}\\ \theta_{y,k+1}\\ \theta_{z,k+1} \end{array}\right]$$ (62)

Under external influence, these matrix equalities turn into matrix inequalities and additional external terms are integrated. Regarding conservation of energy, if this external influence has a known energy relationship, the external work term *W*(***s***) may be seen in Eq. 63. Likewise, the momentum conservation equation is modified with external influences in the form of force and torque, seen in Eq. 64. If rotational degrees of freedom are constrained, the *θ* terms in Eq. 64 are zero, and for constrained translation, the *x*, *y*, *z* terms are zero.

$$\begin{array}{cc} & \frac{1}{2}\left(\left[{\dot{x}}_{k}\;{\dot{y}}_{k}\;{\dot{z}}_{k}\right]M\left[\begin{array}{c} {\dot{x}}_{k}\\ {\dot{y}}_{k}\\ {\dot{z}}_{k} \end{array}\right]+\left[\theta_{xk}\;\theta_{yk}\;\theta_{zk}\right]I\left[\begin{array}{c} \theta_{xk}\\ \theta_{yk}\\ \theta_{zk} \end{array}\right]\right)+U\left(s_{k}\right)+W\left(s_{k}\right)\\ & \;\geq \frac{1}{2}\left(\left[{\dot{x}}_{k+1}\;{\dot{y}}_{k+1}\;{\dot{z}}_{k+1}\right]M\left[\begin{array}{c} {\dot{x}}_{k+1}\\ {\dot{y}}_{k+1}\\ {\dot{z}}_{k+1} \end{array}\right]+\left[\theta_{x,k+1}\;\theta_{y,k+1}\;\theta_{z,k+1}\right]I\left[\begin{array}{c} \theta_{x,k+1}\\ \theta_{y,k+1}\\ \left.\theta_{z,k+1}\right] \end{array}\right]\right)+U\left(s_{k+1}\right)+W\left(s_{k+1}\right) \end{array}$$ (63)

$$M\left[\begin{array}{c} {\dot{x}}_{k}\\ {\dot{y}}_{k}\\ {\dot{z}}_{k} \end{array}\right]+F\left(s_{k}\right)\Delta t+I\left[\begin{array}{c} \theta_{x,k}\\ \theta_{y,k}\\ \theta_{z,k} \end{array}\right]+\tau \left(s_{k}\right)\Delta t=M\left[\begin{array}{c} {\dot{x}}_{k+1}\\ {\dot{y}}_{k+1}\\ {\dot{z}}_{k+1} \end{array}\right]+F\left(s_{k+1}\right)\Delta t+I\left[\begin{array}{c} \theta_{x,k+1}\\ \theta_{y,k+1}\\ \theta_{z,k+1} \end{array}\right]+\tau \left(s_{k+1}\right)\Delta t$$ (64)

Outside of physical laws of dynamics, a more general constraint is a state bound in the form of an inequality. A state boundary could result from a physical boundary, in which a body cannot intersect another body. A lower bound and higher bound are given by Eq. 65, where ***r*** is a reference point.

$$s_{k+1}\leq r\;\text{OR}\;s_{k+1}\geq r$$ (65)

These two constraints can be superimposed as long as the constraints do not conflict with each other. Another set of bounding conditions results from a state relationship and sign of state derivative, given in state inequality (Eq. 66).

$$\begin{array}{c} s_{k+1}\leq s_{k}\;\text{if}\;{\dot{s}}_{k}\leq 0\\ \text{OR}\\ s_{k+1}\geq s_{k}\;\text{if}\;{\dot{s}}_{k}\geq 0 \end{array}$$ (66)

## 7 Solving for state prediction simultaneously with constraints

The proposed NN-Poly method can be used as a system identification method and/or a state prediction method. The state prediction solution in matrix form and in polynomial basis space is given in Eq. 67, where ***y***_*k*_ is either the state derivative ***s***_*k*_ or the propagated state ***s***_*k*+1_.

$$y_{k}={\tilde{J}}_{f}^{d}a^{d}{\tilde{s}}^{⊗d}$$ (67)

As derived previously, ${\tilde{J}}_{f}^{d}$ and ***a*** ^*d*^ are both matrices populated only with numerical coefficients that approximate the NN function. Together, ${\tilde{J}}_{f}^{d}a^{d}$ are an analogous state transition matrix, where the traditional input state ***s***_*k*_ is augmented into the previously defined higher order polynomial basis space ${\tilde{s}}^{⊗d}$ . As previously shown, constraints may be represented as linear equations in the same polynomial basis space. To solve for equality constraints simultaneously with state prediction, the constraint equation matrix, *h*_*e*_, is appended to the polynomial state equation matrix, shown in Eq. 68, and solved with semi-definite programs (SDPs).

$$\left[\begin{array}{c} y_{k}\\ h_{e} \end{array}\right]=\left[\begin{array}{c} {\tilde{J}}_{f}^{d}a^{d}\\ H_{e} \end{array}\right]{\tilde{s}}^{⊗d}$$ (68)

For inequality constraints, numerical programs may be utilized to solve the semi-algebraic optimization problem, given in Eq. 69, where *h*_*i*_ are inequality constraint equations. Solving the semi-algebraic optimization problem is outside scope of this study, but the authors refer us to prevalent tools, such as the linear programming and semi-definite programming packages (Lasserre, 2015). These SDPs are convex but grow arbitrarily large as upon iteration. Future research includes exploiting the information incorporated in constraints known about the system to reduce the size of these SDPs, building upon work of Ahmadi and El Khadir (2020).

$$y_{k}={\tilde{J}}_{f}^{d}a^{d}{\tilde{s}}^{⊗d}\;\text{such that}\;h_{i}\leq H_{i}{\tilde{s}}^{⊗d}$$ (69)

One can implement constrained state prediction in real-time on an embedded dynamic system in a learning application. In an algorithmic loop, a neural network may be trained with every iteratively collected measurement, then the subsequent polynomial may be generated and the state prediction/control effort constrained with predefined constraints. Other applications include post-processing system identification for data-constrained applications, in which neural network parameters are more effective to communicate than large data sets, like space or deep-sea applications.

## 8 NN-Poly state prediction performance

Our aim in these results is to show the minimal approximation loss between the NN-Poly and the models that were derived directly from data: the neural network and polynomial. In the unconstrained case, the NN-Poly can only be as accurate as the neural network from which the polynomial is derived from as the polynomial approximates the neural network. Similarly, the NN-Poly can only be as accurate as a polynomial found from the original data because NN-Poly lost information between the original data and the neural network representation. In the constrained case, the NN-Poly has the ability to incorporate domain knowledge that the neural network cannot capture, which offers NN-Poly the ability to better represent the underlying dynamic system generating the data; for example, a neural network trained on data generated from a bouncing ball may predict the ball’s state to intersect with the ground, but constraints placed on the polynomial model would bound predictions such that the ball never intersects with the ground, yielding a more accurate state prediction. The proposed Taylor expansion with the NN parameters is coded in MATLAB (https://github.com/alexdhjing/NNX_matlab). Metrics to evaluate performance of each method are efficiency, accuracy, and complexity. This section discusses the method of comparison, metrics to evaluate performance, and subsequent results of the state prediction methods.

### 8.1 Methods

Several dynamic models are used to measure efficiency and accuracy of each state prediction method, not only in the state prediction but also in the model representation. Each algorithm is trained with the same pairwise input and output data, generated from the true dynamic model, to predict a state for a range of dynamic systems. The input–output data pairs are of form {***x***_*k*_, ***x***_*k*+1_}, in which the input is state vector at time step *k* and the output is of time *k* + 1. A majority of data pairs train the various models and the rest of the data pairs are set aside to evaluate state error and computation time. Other metrics evaluate the model coefficients and structure after training. All metrics are formally defined in the next subsection.

The selected dynamic models were chosen in ascending complexity, from a two-dimensional feature space with linear dynamics and non-linear dynamics. The 1DOF underdamped linear spring mass damper system has a linear transition matrix from previous state to next state, constituting the simplest dynamic system to identify, described in Eq. 70. The 1DOF non-linear spring system has non-linear spring stiffness and is governed by a differential equation, given in Eq. 71. A 2DOF spring pendulum demonstrates non-linearity and coupling of dynamics, given in Eq. 72. In both the 1DOF non-linear spring and 2DOF spring pendulum cases, the differential equations of motion propagate the state to generate data. The system identification methods do not generate expressions for acceleration but generate a mapping from previous state to next state, implicitly integrating the double derivative.

$$\left[\begin{array}{c} x_{t+1}\\ v_{t+1} \end{array}\right]=\left[\begin{array}{cc} 0.9995 & 0.01\\ -0.0999 & 0.9985 \end{array}\right]\left[\begin{array}{c} x_{t}\\ v_{t} \end{array}\right]$$ (70)

$$\ddot{x}=\frac{-3\mu_{0}}{2\pi {\left(x+a\right)}^{4}}$$ (71)

$$\left[\begin{array}{c} \ddot{x}\\ \ddot{y} \end{array}\right]=\left[\begin{array}{c} -5\left(\sqrt{x^{2}+y^{2}}-1\right)\frac{x}{\sqrt{x^{2}+y^{2}}}\\ -5\left(\sqrt{x^{2}+y^{2}}-1\right)\frac{y}{\sqrt{x^{2}+y^{2}}}-10 \end{array}\right]$$ (72)

### 8.2 Metrics of evaluation

Metrics for evaluation are computation time, state error (mean squared error), coefficient error, length of solution, and coefficient stability, given in Table 2. The state error is of mean squared error form of the algorithm’s state prediction and the true state for which smaller error represents a more accurate approximation. Coefficient error refers to a coefficient vector ***c*** of length *m*, geared toward polynomial solutions for which smaller error again signifies a more accurate model. Parameter stability refers to the learned parameters, relevant for all methods, for which values closer to 1 represent high stability and values approaching infinity represent instability. The length of solution refers to the number of *h*(***x***) for which a smaller number of terms represents a more parsimonious solution.

**TABLE 2:** Metrics for evaluating system identification methods on dynamic systems.

Metrics	Measurement	Definition/equation
Computing time	Efficiency	Time cost to run calculation
State error (mean squared error)	State accuracy	$MSE=\frac{1}{T}\sum_{t=1}^{T}{\left(x_{k}-{\hat{x}}_{k}\right)}^{2}$
Coefficient error	Expression accuracy	MSE of coefficients (if in polynomial form) $CE=\frac{1}{m}\sum_{i=1}^{m}{\left(c_{i}-{\hat{c}}_{i}\right)}^{2}$ Marked as N/A if not in polynomial form
Parameter stability	Expression accuracy and complexity	Largest parameter divided by smallest coefficient $PS=\frac{\max\left(p\right)}{\min\left(p\right)}$
Length of solution	Expression accuracy and complexity	Number of nonlinear terms in solution

### 8.3 Complexity

We will explore the complexity of our proposed method by deriving and comparing the number of flops for the following methods: a polynomial directly from data, training a NN and NN-Poly. Intuitively before even approaching a rigorous derivation, the NN-Poly method explicitly relates NN parameters to polynomial coefficients, emulating a “hard-coded” computation. We added computing time into the evaluation metrics as calling table elements from memory would likely be the most computationally intensive task in the NN-Poly method. Calculating polynomial coefficients directly from a large data set with the least-squares method will be computationally intensive due to the matrix inverse operation that scales cubed with dimension of the data set. A neural network’s flops depend on training epochs until convergence, stochastically related to the initial parameterization of the neural network. For all flop calculations, we will assume that:

• The input state size *m* and output state size *n* are equivalent *m* = *n*
• The state size *m* is much smaller than the amount of training points *t*, *m* ≪ *t*

The least-squares method to calculate polynomial coefficients *A* with a matrix of inputs *X* and outputs *Y* is given in Eq. 73, where *A*_*p*_ is the set of polynomial coefficients derived from the least-squares method.

$$A_{p}=Y{\left(X^{⊗d}\right)}^{T}{\left(\left(X^{⊗d}\right){\left(X^{⊗d}\right)}^{T}\right)}^{-1}$$ (73)

The number of flops of a polynomial derived directly from data is on the order given in Eq. 74. Intermediate steps are given in Eqs 135–138 in Supplementary Appendix Section S10.5.

$$O\left(A_{p}\right)\approx t\frac{m^{d}}{d!}+\frac{m^{3d}}{d!}$$ (74)

To train neural networks, we must account for forward passes and backward propagation per epoch overall training data. In implementation, the neural network does not necessarily train across all the data but for this derivation of flops will assume so to simplify the calculation. We will also assume that the network has only a single layer with *k* neurons. The total number of flops to train a neural network is given in Eq. 75.

$$O\left(\text{NN}\right)\approx mnkt$$ (75)

To convert this neural network into a polynomial, the polynomial coefficients for each polynomial degree scales with the number of neurons in the layer. The highest order polynomial dominates the number of flops, which approximates the total number of flops in converting a single-layer NN with *k* neurons to a polynomial of degree *d* given in Eq. 76. The set of polynomial coefficients derived from the NN is given by $A_{NN}^{d}$ . Intermediate steps are shown in Eqs 140–145 in Supplementary Appendix Section S10.5.

$$O\left(A_{NN}^{d}\right)\approx km^{d+1}$$ (76)

The combined complexity of training a neural network and converting those NN parameters to polynomial coefficients is given in

$$O\left(\text{NN}+A_{NN}^{d}\right)\approx km^{2}t+km^{d+1}$$ (77)

$$=k\left(m^{2}t+m^{d+1}\right)$$ (78)

The condition for the neural network training to be more computationally complex than the NN-Poly conversion is given in Eq. 79.

$$\left\{\begin{array}{cc} O\left(\text{NN}\right)>O\left(A_{NN}^{d}\right),\; & \text{if }t>m^{d-1}\\ O\left(A_{NN}^{d}\right)>O\left(\text{NN}\right),\; & \text{otherwise} \end{array}\right.$$ (79)

Given the order of complexity of each method that ultimately yields a polynomial (least-squares polynomial directly from data and polynomial from NN parameters), what are the conditions on state size *m*, training data size *t*, and neural network size by neurons *k* that make either method more or less complex than the other? The condition for the NN-Poly method to be less complex than a polynomial from the raw data is given in Eq. 80.

$$\left\{\begin{array}{cc} O\left(A_{p}\right)>O\left(\text{NN}+A_{NN}^{d}\right),\; & \text{if }mk<\frac{t+m^{d}}{d!}\\ O\left(\text{NN}+A_{NN}^{d}\right)>O\left(A_{p}\right),\; & \text{otherwise} \end{array}\right.$$ (80)

Intuitively, the NN-Poly method is computationally advantageous for the following conditions:

• There are many training points
• The neural network is small
• The state size is large
• The state size is sufficiently large and the polynomial degree is large

Complexity does not fully capture computational load though, as the NN-Poly method relies heavily on memory calls. The next section incorporates computing time for this reason.

### 8.4 Results

For each dynamic system, Tables 3–5 report the performance of the strict polynomial, the sole NN and NN-Poly for each dynamic system. Wherever an *ϵ* is listed in the tables, *ϵ* denotes an immensely small value within machine precision. The computation time for the NN-polynomial expansion does not include the NN training time, only the time to transform the NN parameters into polynomial coefficients.

**TABLE 3:** 1DOF spring evaluation of performance for all system identification methods.

Metrics	Polynomial	NN	NN-Poly
Computing time	**0.29 s**	3.63 s	0.37 s
State error (MSE)	*ϵ*(*x*)	**1.1** × 10^–8^(*x*)	1.1 × 10^–5^(*x*)
	*ϵ*(*x*)	**1.5** × 10^–8^(*v*)	1.5 × 10^–5^(*v*)
Coefficient error (CE)	0.006 (x)	N/A	**0 (x)**
	0.006 (v)		**0 (v)**
Parameter stability	99.95 (x)	99.95(*x*)	99.95(*x*)
	9.95 (v)	9.995(*v*)	9.995(*v*)
Number of parameters	6	6	6

Bolded values are the best values across methods for each metric.

**FIGURE 3:**
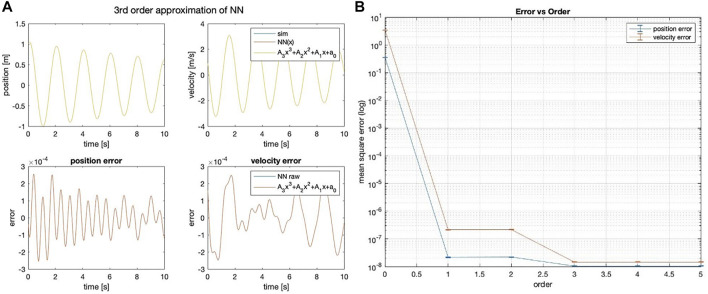
**(A)** Third-order NN-Poly approximation of 1DOF linear system and error as difference of each state. **(B)** MSE error of position and velocity state across different orders of approximation for NN-Poly.

The method that best predicts the 1 DOF linear spring state is the strict polynomial; although curiously, the NN-Poly exactly predicts the original system state matrix coefficients. The performance across all methods is shown in Table 3. The proposed NN-Poly approximation method performance in each state’s time series and across increasing polynomial degrees is shown in Figure 3. As expected, increasing the order of polynomial approximation yields less state error, although not much performance is gained past the second-order approximation, seen in Figure 3. The small error bars for each degree signals that the final solution from each NN random initialization does not vary error much. The NN-polynomial conversion is comparable in computation but includes more state error as expected. This straightforward test case is best solved with the simplest solver, the direct polynomial solution; even a least squares solution is sufficient. This system does not need a complex, expressive system identification method due to the simplicity of its linear form.

**FIGURE 4:**
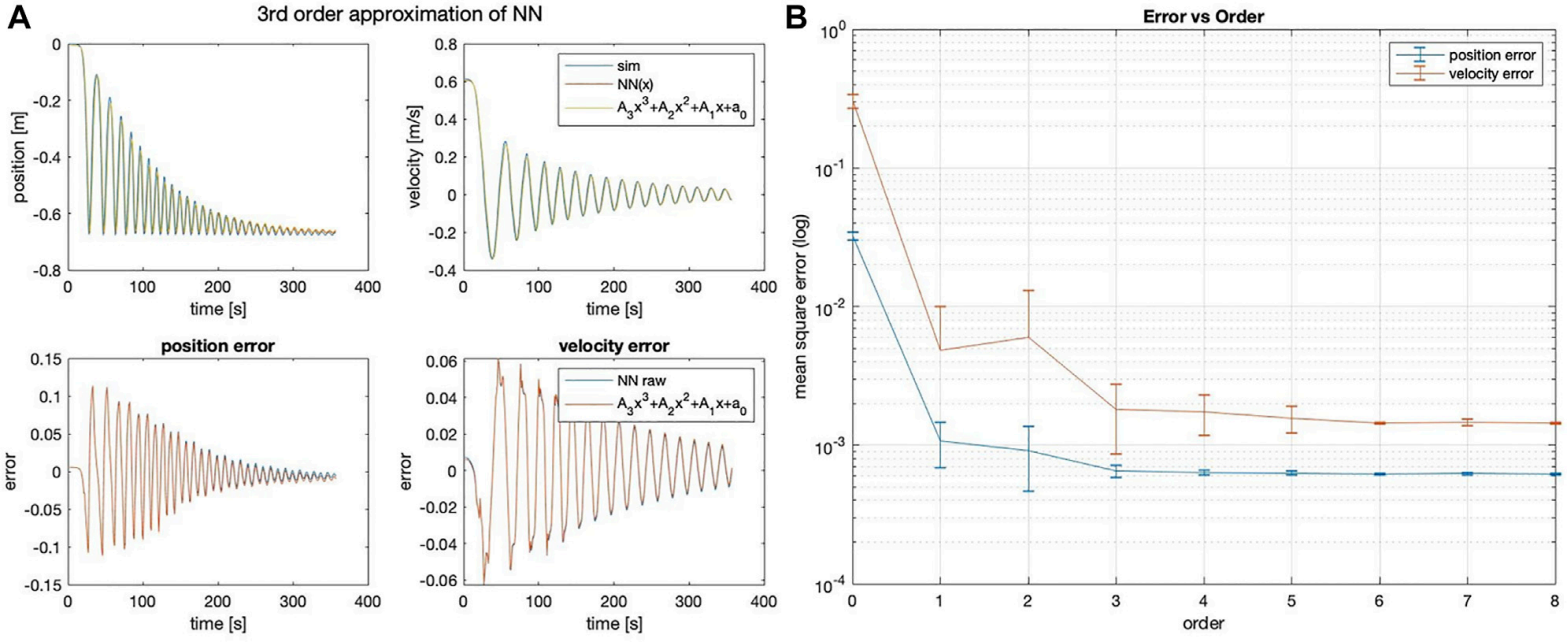
**(A)** Third-order NN-Poly approximation of 1DOF non-linear spring system and error as difference of each state. **(B)** MSE error of position and velocity state across different orders of approximation for NN-Poly.

The method that best predicts the 1 DOF non-linear spring state are NN and NN-Poly, with performance reported in Table 4. Unlike the linear system approximation, the proposed NN-Poly approximation varies from the NN parameter initialization and does not converge to a steady state error until the fourth order, seen in Figure 4. The sole NN and NN-Poly approximate the data with the least state error, generate a minimal representation, and yield the most stable parameters of all methods. Subsequently, the NN-Poly retains the same value of state error from NN, demonstrating the accuracy of transformation from a NN form to polynomial form, while also offering a form from which to apply domain knowledge and safety guarantees.

**TABLE 4:** 1 DOF flux-pinned system evaluation of performance for all system identification methods.

Metrics	Polynomial	NN	NN-Poly
Computing time	**0.076 s**	1.36 s	0.20 s
State error (MSE)	1.9, ×, 10^–3^(*x*)	**1.3** × 10^–4^(*x*)	**1.3** × 10^–4^(*x*)
	9.0, ×, 10^–4^(*v*)	**1.1** × 10^–4^(*v*)	**1.1** × 10^–4^(*v*)
Parameter stability	5.8 × 10^4^(*x*)	**67.8(x)**	94.8 (x)
	2.8 × 10^3^(*v*)	**574(v)**	579.4 (v)
Number of parameters	30	**6**	**6**

Bolded values are the best values across methods for each metric.

**FIGURE 5:**
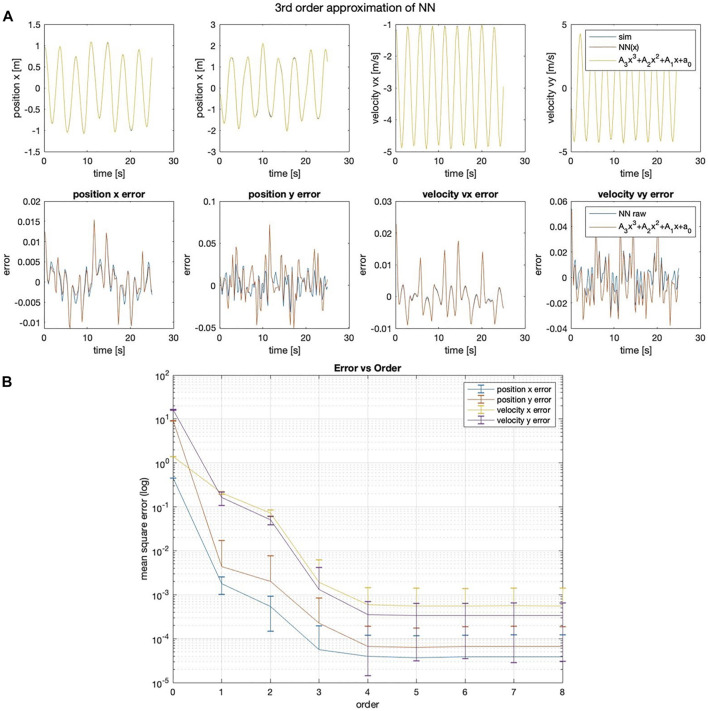
**(A)** Third-order NN-Poly approximation of a 2DOF non-linear coupled spring–pendulum system and error as difference of each state. **(B)** 2DOF spring–pendulum individual MSE error of position and velocity state across different orders of NN-Poly approximation.

For the 2DOF system, NN and NN-Poly predict state accurately, seen in Table 5. The third order NN-poly approximation for each state is shown in Figure 5A. The NN-Poly MSE error decreases with increasing polynomial degree and does not converge until after the fourth order, seen in Figure 5B. The NN and NN-Poly predict the 2DOF system with fewer terms. Each method has comparable parameter stability and computation time. This coupled and slightly non-linear dynamic system straddles the boundary in deciding which model to use.

**TABLE 5:** 2 DOF spring pendulum system evaluation of performance for all system identification methods.

Metrics	Polynomial	NN	NN-Poly
Computing time	**0.29 s**	3.58 s	0.36 s
State error (MSE)	**5.0** × 10^–4^(*x*)	3.0 × 10^–3^(*x*)	3.2 × 10^–3^(*x*)
	**3.5** × 10^–3^(*v*)	1.62 × 10^–2^(*v*)	1.7 × 10^–2^(*v*)
Parameter stability	**6.6** × 10^3^(*x*)	7.6 × 10^3^(*x*)	8.1 × 10^3^(*x*)
	**8.6** × 10^3^(*v*)	1.3 × 10^3^(*v*)	1.4 × 10^3^(*v*)
Number of parameters	120	**20**	140

Bolded values are the best values across methods for each metric.

## 9 Conclusion

Leveraging recent advances in deep learning, NN-Poly provides accurate predictions of non-linear, coupled system dynamics with minimal context. A NN-to-polynomial mapping avoids the need to download an immense amount of data and fit a polynomial directly to a large dataset by exploiting the compactness of the NN. A polynomial enhances interpretability of the neural network by making its feature dependence explicit, as polynomials have a long history of analysis and mathematical literature, including safety verification and guarantees. This work’s major contribution is offering a polynomial form and semi-algebraic constraints, such polynomial inequality and equality constraints, to capture the NN model and system context in the final predictive function solution. These semi-algebraic constraints represent the application of domain knowledge, such as conservation of energy in the form of quadratic rates, and safety constraints, such as linear inequalities that bound specific state values. The results of this effort show comparable prediction and computation performance between a sole NN, sole polynomial, and the proposed method for linear systems but great improvement in the proposed method for highly nonlinear systems. Further future work also includes approximation of more complex systems, in increasing degrees of freedom, in the degree of non-linearity, and in the coupling of states.

## Data Availability

The datasets presented in this study can be found in online repositories. The names of the repository/repositories and accession number(s) can be found as follows: https://github.com/alexdhjing/NNX_matlab.
